# Marginalized Neighborhoods and Health Outcomes in Younger Myocardial Infarction Survivors

**DOI:** 10.1001/jamanetworkopen.2025.18826

**Published:** 2025-07-02

**Authors:** Leo E. Akioyamen, Atul Sivaswamy, Olivia Haldenby, Husam Abdel-Qadir, Maneesh Sud, David A. Alter, Clare L. Atzema, Peter C. Austin, Cynthia A. Jackevicius, Moira K. Kapral, Harlan M. Krumholz, Karen Tu, Harindra C. Wijeysundera, Dennis T. Ko

**Affiliations:** 1 Department of Medicine, University of Toronto, Toronto, Ontario, Canada; 2 ICES, Toronto, Ontario, Canada; 3 Institute of Health Policy, Management, and Evaluation, University of Toronto, Toronto, Ontario, Canada; 4 University Health Network, Toronto, Ontario, Canada; 5 Women’s College Hospital, Toronto, Ontario, Canada; 6 Schulich Heart Program, Sunnybrook Health Sciences Centre, Toronto, Ontario, Canada; 7 Western University of Health Sciences, Pomona, California; 8 Center for Outcomes Research and Evaluation, Yale New Haven Hospital, New Haven, Connecticut; 9 Section of Cardiovascular Medicine, Department of Internal Medicine, Yale School of Medicine, New Haven, Connecticut; 10 Department of Health Policy and Management, Yale School of Public Health, New Haven, Connecticut; 11 North York General Hospital, Toronto, Ontario, Canada; 12 Department of Family and Community Medicine, University of Toronto, Toronto, Ontario, Canada

## Abstract

**Importance:**

Neighborhood characteristics may be independently associated with survival after acute myocardial infarction (AMI).

**Objective:**

To examine the association of living in a marginalized neighborhood with mortality and care for younger AMI survivors (aged <65 years) in a universal health care system.

**Design, Setting, and Participants:**

Population-based retrospective cohort using clinical and administrative databases in Ontario, Canada. Participants were younger patients hospitalized for their first AMI who received invasive evaluation and survived to 7 days after discharge between April 1, 2010, and March 1, 2019. Statistical analysis was performed between May 27, 2022, and March 31, 2025.

**Exposures:**

Neighborhood marginalization, a metric comprising material deprivation, residential instability, and dependency.

**Main Outcomes and Measures:**

All-cause death, all-cause hospitalizations, and subsequent AMIs. Proportional hazards regression models were used to quantify the association of marginalization with outcomes over 3 years.

**Results:**

Among 65 464 AMI patients (median age, 56 [IQR, 50-61] years; 22.9% female), increasing neighborhood marginalization was associated with higher rates of mortality beginning 30 days after discharge and persisting over time. At 3 years, mortality rates ranged from 2.2% in the least marginalized neighborhood quintile (Q1) to 5.2% in the most marginalized (Q5). Adjusted hazard ratios for mortality over 3 years of follow-up were significantly higher in patients from marginalized neighborhoods and ranged from 1.13 (95% CI, 0.95-1.35) in Q2 to 1.52 (95% CI, 1.29-1.80) in Q5. Over 1 year, differences were observed between Q1 and Q5 in visits to primary care physicians (Q1, 96.1%; Q5, 91.6%) and cardiologists (Q1, 88.0%; Q5, 75.7%), as well as diagnostic testing.

**Conclusions and Relevance:**

In this cohort study of younger AMI survivors with universal health care, living in marginalized neighborhoods was associated with adverse outcomes. The observed differences in health service utilization among marginalized patients warrant further investigation to better understand the underlying structural and systemic factors.

## Key Points

**Question:**

What is the association of living in a marginalized neighborhood with mortality and care for younger survivors of acute myocardial infarction (AMI) in a universal health care system?

**Findings:**

In this cohort study including 65 464 patients with AMI, increasing neighborhood marginalization was associated with higher rates of mortality, hospitalizations, and recurrent myocardial infarction beginning 30 days after discharge that persisted over time. Lower rates of physician care and diagnostic testing were observed despite universal health care coverage.

**Meaning:**

Living in a marginalized neighborhood was associated with adverse outcomes and less care among younger AMI survivors; observed differences in health service use warrant further investigation to uncover underlying systemic drivers.

## Teaser

This cohort study assesses the association between living in a marginalized neighborhood and outcomes in younger patients (<65 years old) who survived acute myocardial infarction in a universal health care system.

## Introduction

The neighborhood in which a person lives is an important determinant of their cardiovascular health.^1^ Neighborhood socioeconomic environments have been shown to be related to important health behaviors such as smoking, dietary habits, and physical activity.^2^ Additionally, neighborhood socioeconomic disadvantage has been linked to greater incidence of coronary heart disease and associated mortality.^3,4,5^ Characteristics of neighborhoods appear to affect health outcomes independent of the personal socioeconomic positions of individuals.^5^ However, most studies of neighborhood impacts in health outcomes originate from settings allowing private payments for health care,^3,4,5^ making it challenging to separate the effects of neighborhoods from those of insurance status and the inability to pay.

Younger adults (aged <65 years) represent a growing proportion of those with acute myocardial infarction (AMI),^6,7,8,9^ making cardiovascular disease one of the leading causes of premature morbidity and death.^10^ The burden of coronary artery disease is expected to increase as modifiable risk factors become more prevalent in the younger population.^11,12^ Consequently, the cardiology community has prioritized identifying underlying drivers of cardiovascular disease in this age group.^13^ Younger adults may be more vulnerable to the impacts of adverse neighborhood conditions in cardiovascular health.^14^ Moreover, differences between neighborhoods may be the result of policies directly amenable to intervention to reduce mortality and promote health.^1,15^ Yet, few studies to date have explored the association of neighborhood socioeconomic characteristics with AMI outcomes in younger adults.^3,16,17,18^

In this study, we assessed the association between living in a marginalized neighborhood and outcomes in younger patients who survived AMI in a universal health care system. We used the Ontario Marginalization Index,^19,20^ a multidimensional area-based measure of socioeconomic status composed of material deprivation, residential instability, and dependency.

## Methods

This retrospective cohort study used population-wide data from a clinical registry linked with administrative databases via unique encoded patient identifiers and was analyzed at ICES.^21^ The data used in this project was authorized under section 45 of Ontario’s Personal Health Information Protection Act, which does not require review by a research ethics board. This study was conducted in accordance with the Strengthening the Reporting of Observational Studies in Epidemiology (STROBE) reporting guideline for cohort studies.^22^

### System Context

Ontario is Canada’s most populous province, with more than 15 million residents. A publicly funded health insurance system provides universal access to medical and surgical physician services for all permanent residents, with no user fees at the point of service. There are currently 20 hospitals with the capability to perform invasive cardiac procedures that provide services for more than 130 hospitals in the province.

### Data Sources

Our study leveraged clinical data from the CorHealth Ontario Cardiac Registry,^23,24^ which collects demographic, clinical, laboratory, and procedural characteristics of all adult patients undergoing invasive cardiac procedures in Ontario. Data included in the registry are recorded by referring physicians at the time of coronary angiography referral while trained individuals subsequently abstract details of coronary anatomy and interventions.

We linked the CorHealth database with (1) the Registered Persons Database, which contains vital statistics; (2) the Canadian Institute for Health Information Discharge Abstract Database, which captures hospitalization events; (3) the National Ambulatory Care Reporting System, which collects data on emergency department visits; (4) the Ontario Health Insurance Plan database, which records physician billing claims to capture visits of patients to physicians; (5) the ICES Physician database, which was used to determine the per capita supply of cardiologists within the province; and (6) the Ontario Marginalization Index, which was used to derive neighborhood-level marginalization measures.

### Cohort Creation

Our study cohort included adults younger than 65 years who had their first hospitalization for AMI (*International Statistical Classification of Diseases and Related Health Problems, Tenth Revision* codes I21 or I22) between April 1, 2010, and March 1, 2019. Statistical analysis was performed between May 27, 2022, and March 31, 2025. To examine the association between the neighborhood environment and patients receiving contemporary AMI care, we included patients who received cardiac catheterization during their hospitalization and survived to 7 days after discharge.^22^ To ensure all admissions were for a first AMI and minimize confounding due to prior cardiac interventions,^22^ we excluded patients with previous percutaneous coronary intervention or coronary artery bypass grafting within 5 years before their index hospitalization. Additionally, for patients who had multiple hospitalizations for AMI during our study period, we counted the first admission as the index event for study inclusion. Patients transferred between hospitals were also included. We excluded patients who were not residents of Ontario, those who were residents of long-term care facilities at the time of index hospital admission, and patients missing information necessary for data linkage (age, sex, Ontario Health Insurance Plan number, and Ontario Marginalization Index scores). Finally, we did not include patients with length of stay shorter than 1 day or those for whom AMI was encountered as an in-hospital complication.^22^

### Determining Marginalization

Our primary exposure was neighborhood marginalization, assessed at baseline using the Ontario Marginalization Index.^19,20^ This validated area-based measure provides a multidimensional estimate of the degree of marginalization experienced by residents of different neighborhoods. These dimensions are derived from analysis of sociodemographic variables from the Canadian Census of Population previously identified as factors associated with inequality.^19^ Similar to prior work,^25,26,27^ we defined marginalization using a composite measure of material deprivation, residential instability, and dependency (eTable 1 in Supplement 1). We combined the 3 select dimensions of marginalization to create summary scores and then divided by 3 to produce a composite measure of marginalization. We then used the composite measure to subdivide patients into quintiles that represent increasing levels of marginalization from the least (Q1) to the most marginalized (Q5).

### Outcomes

Our primary outcome was all-cause death. Additionally, we examined adverse events following the index hospitalization, which were subsequent AMI hospitalizations and hospitalizations for any cause. Ascertainment of outcomes began at 7 days following hospital discharge and continued until the occurrence of the primary outcome or the end of the study follow-up. All outcomes evaluated were examined within 30 days, 1 year, and 3 years using separate models.

We also examined processes of clinical care as secondary outcomes at 30 days and 1 year, which included visits to primary care physicians and cardiologists, diagnostic tests (ie, echocardiograms, ambulatory electrocardiogram monitoring, and stress tests), and invasive evaluations and treatment (coronary angiography, coronary revascularization that comprised percutaneous coronary intervention or coronary artery bypass grafting).

### Statistical Analysis

AMI patients were categorized by quintile of neighborhood marginalization. We individually compared outcomes among patients living in neighborhoods with progressively higher levels of marginalization (ie, second, third, fourth, and fifth quintiles), with the first quintile serving as the reference group. We calculated descriptive statistics for the cohort’s sociodemographic characteristics, cardiovascular risk factors, and other comorbid diseases. Categorical variables were expressed as percentages (with counts) and compared using χ^2^ tests, while continuous variables were reported as medians (quartiles) and compared using the Kruskal-Wallis test. We used proportional hazards regression models to determine the association of marginalization quintiles with all-cause mortality and hospitalizations. We used a robust variance estimator to account for the clustering of patients in enumeration areas or dissemination areas, which were the physical neighborhoods in which marginalization was measured. The proportional hazards regression assumption was evaluated using graphical methods and a likelihood ratio test that showed no significant violation.

Multivariable models were adjusted for demographic characteristics (age, sex, rural residence, the proportion of individuals identifying as a recent immigrant or visible minority), cardiac risk factors (hypertension, diabetes, dyslipidemia, and smoking status), disease severity on index presentation (ST-segment myocardial infarction [STEMI], left ventricular function, and revascularization procedure received), cardiovascular and noncardiac comorbidities (peripheral or cerebral vascular disease, prior hospitalizations for congestive heart failure, asthma, chronic obstructive pulmonary disease, chronic kidney disease, frailty,^28^ dementia, history of prior cancer, and Charlson Comorbidity Index score), and per capita supply of cardiologists. We report hazard ratios (HRs) and 95% CIs for all outcomes of interest; *P* values were not reported given our study’s large sample size. Analyses were performed using SAS Enterprise Guide, version 8.3 (SAS Institute Inc).

## Results

### Creation of the Study Sample

**Figure 1.**
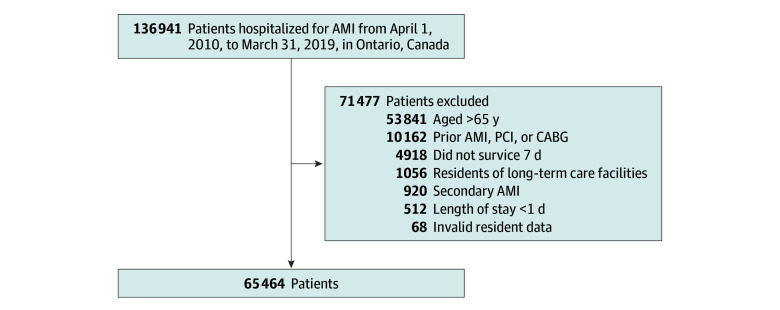
Cohort Creation AMI indicates acute myocardial infarction; CABG, coronary artery bypass graft; PCI, percutaneous coronary intervention.

We identified 136 941 patients who received cardiac catheterization during their first AMI admission (Figure 1). We excluded 53 841 patients older than 65 years, 10 162 with previous AMIs or revascularization, 4918 who died within 7 days of hospital discharge, 1056 residents of long-term care facilities, 920 patients with a secondary AMI, 512 who had a length of stay shorter than 1 day, and 68 patients with invalid data. This left 65 464 patients for inclusion.

### Baseline Characteristics

The median age for the overall cohort was 56 (IQR, 50-61) years and 14 968 patients (22.9%) were female (Table 1). Patients in the most marginalized quintiles had a greater proportion of females (Q1, 18.1%; Q5, 27.2%) and were more likely to live in rural areas. They were also more likely to have preexisting cardiac risk factors and noncardiac comorbidities. The rate of active smoking was also substantially higher among patients in the most marginalized quintile (Q1, 31.3%; Q5, 48.9%). At hospital presentation, patients in the most marginalized quintile were least likely to present with a STEMI (Q1, 49.6%; Q5, 47.5%). There were no differences in presentation with triple-vessel disease or impaired left ventricular function. The most marginalized patients had the lowest rates of percutaneous coronary intervention (Q1, 76.0%; Q5, 71.5%) and equivalent rates of coronary artery bypass grafting (Q1, 1.3%; Q5, 1.2%) during the index admission.

**Table 1.** Baseline Characteristics of Individuals by Quintile of Marginalization of Individuals’ Residence

Characteristic	Patients by quintile of marginalization, No. (%)				
	Quintile 1 (least marginalized) (n = 9654)	Quintile 2 (n = 10 916)	Quintile 3 (n = 13 030)	Quintile 4 (n = 15 899)	Quintile 5 (most marginalized) (n = 15 965)
Proportion of all patients, %	14.7	16.7	19.9	24.3	24.4
Age, median (IQR), y	55 (49-60)	56 (50-61)	56 (51-61)	56 (50-61)	56 (50-61)
Men	7905 (81.9)	8769 (80.3)	10 161 (78.0)	12 042 (75.7)	11 619 (72.8)
Women	1749 (18.1)	2147 (19.7)	2869 (22.0)	3857 (24.3)	4346 (27.2)
Rural residence	602 (6.2)	1430 (13.1)	2064 (15.8)	2261 (14.2)	2634 (16.5)
Cardiovascular disease risk factors					
Hypertension	5786 (59.9)	6607 (60.5)	8089 (62.1)	10 141 (63.8)	10 317 (64.6)
Diabetes	2483 (25.7)	2839 (26.0)	3573 (27.4)	4683 (29.5)	4904 (30.7)
Hyperlipidemia	4826 (50.0)	5317 (48.7)	6387 (49.0)	7913 (49.8)	7851 (49.2)
BMI	28 (25-32)	28 (25-32)	28 (25-32)	28 (25-32)	28 (25-33)
Smoking^a^					
Active	3017 (31.3)	3846 (35.2)	5187 (39.8)	6940 (43.7)	7802 (48.9)
Former	1562 (16.2)	1860 (17.0)	2208 (16.9)	2647 (16.6)	2773 (17.4)
Preexisting comorbidities^b^					
Asthma	1176 (12.2)	1387 (12.7)	1631 (12.5)	2269 (14.3)	2443 (15.3)
Obstructive pulmonary disease	1019 (10.6)	1488 (13.6)	2117 (16.2)	2926 (18.4)	3643 (22.8)
Cancer	208 (2.2)	261 (2.4)	340 (2.6)	416 (2.6)	462 (2.9)
Cardiovascular disease	226 (2.3)	292 (2.7)	365 (2.8)	566 (3.6)	674 (4.2)
Peripheral vascular disease	201 (2.1)	249 (2.3)	388 (3.0)	581 (3.7)	722 (4.5)
Heart failure	683 (7.1)	766 (7.0)	1070 (8.2)	1455 (9.2)	1669 (10.5)
Dementia	13 (0.1)	28 (0.3)	33 (0.3)	45 (0.3)	63 (0.4)
Anemia	182 (1.9)	215 (2.0)	285 (2.2)	397 (2.5)	429 (2.7)
Chronic kidney disease	115 (1.2)	175 (1.6)	223 (1.7)	329 (2.1)	409 (2.6)
Dialysis	91 (0.9)	112 (1.0)	134 (1.0)	193 (1.2)	220 (1.4)
High frailty risk	22 (0.2)	40 (0.4)	54 (0.4)	86 (0.5)	129 (0.8)
Charlson Comorbidity Index score, mean (SD)	1.6 (1.0)	1.6 (1.1)	1.6 (1.2)	1.7 (1.2)	1.8 (1.3)
Index presentation					
ST-elevation myocardial infarction	4788 (49.6)	5337 (48.9)	6232 (47.8)	7756 (48.8)	7578 (47.5)
Coronary anatomy					
Left main disease	305 (3.2)	382 (3.5)	489 (3.8)	573 (3.6)	669 (4.2)
Triple-vessel disease	1324 (13.7)	1527 (14.0)	1753 (13.5)	2211 (13.9)	2206 (13.8)
Left ventricular function^c^					
<20%	56 (0.6)	66 (0.6)	82 (0.6)	112 (0.7)	133 (0.8)
20%-34%	673 (7.0)	722 (6.6)	868 (6.7)	1115 (7.0)	1117 (7.0)
35%-49%	2320 (24.0)	2601 (23.8)	2900 (22.3)	3507 (22.1)	3531 (22.1)
≥50%	5060 (52.4)	5637 (51.6)	6712 (51.5)	8063 (50.7)	8178 (51.2)
Hospital treatment					
Percutaneous coronary intervention	7339 (76.0)	8162 (74.8)	9478 (72.7)	11 740 (73.8)	11 416 (71.5)
Coronary artery bypass grafting	123 (1.3)	171 (1.6)	151 (1.2)	196 (1.2)	197 (1.2)

Abbreviation: BMI, body mass index (calculated as weight in kilograms divided by height in meters squared).

^a^ Data on smoking status was unknown or missing in 5.7% of patients; 36.5% of patients had never smoked.

^b^ Comorbid diseases were captured prior to admission.

^c^ Left ventricular function missing in 18.3% of patients.

### Mortality and Adverse Events Following Index AMI Hospitalization

Our study cohort had overall crude 30-day, 1-year, and 3-year mortality rates of 0.4%, 1.6%, and 3.8%, respectively. Following the index AMI hospitalization, mortality and adverse events occurred with a gradient of increasing risk with increasing neighborhood marginalization that began at 30 days after discharge and persisted or worsened over time (eTable 2 in Supplement 1). After adjustment for confounders (eFigure in Supplement 1), patients in the most marginalized quintile had significantly greater hazards of all-cause death over 30 days (HR, 2.43; 95% CI, 1.41-4.18), and hospitalization from all causes (HR, 1.16; 95% CI, 1.05-1.28) but not recurrent AMI.

**Figure 2.**
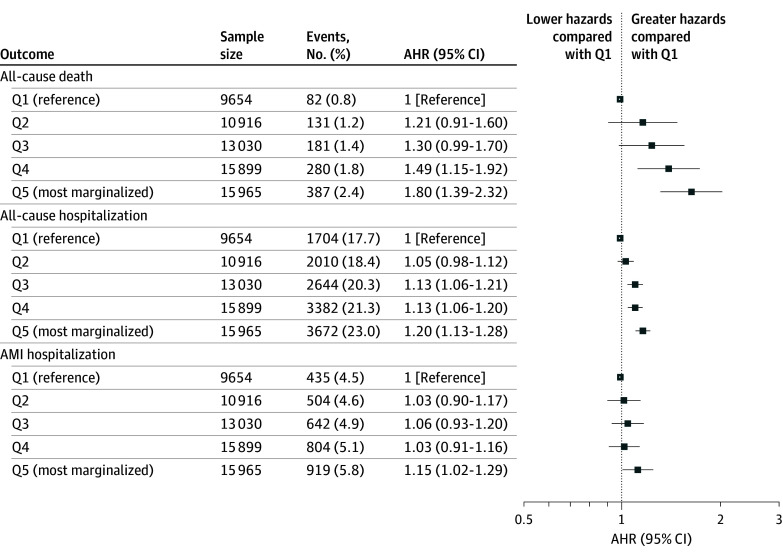
Adverse Events Within 1 Year of Index Acute Myocardial Infarction Hospitalization by Marginalization Quintile (Q) Hazard ratios are relative to patients living in neighborhoods in the first quintile (least marginalized) and are adjusted for demographic characteristics, cardiac risk factors, disease severity on index presentation, comorbid diseases, and per capita concentration of cardiologists. AHR indicates adjusted hazard ratio; AMI indicates acute myocardial infarction.

One year following the index AMI, there were gradients of increasing rates of adverse outcomes with progressively higher marginalization. Patients from Q5 demonstrated the highest adjusted hazard ratios (AHR) of all-cause death (AHR, 1.80; 95% CI, 1.39-2.32) and hospitalization from all causes (AHR, 1.20; 95% CI, 1.13-1.28). While this gradient was not observed for the hazards of a subsequent AMI, patients from Q5 did demonstrate greater hazards over 1 year (AHR, 1.15; 95% CI, 1.02-1.29) (Figure 2).

**Figure 3.**
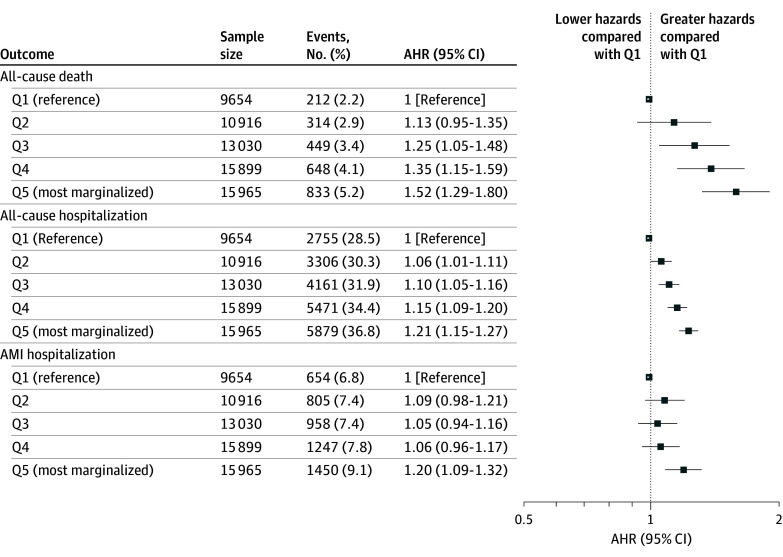
Adverse Events Within 3 Years of Index Acute Myocardial Infarction Hospitalization by Marginalization Quintile (Q) Hazard ratios are relative to patients living in neighborhoods in the first quintile (least marginalized) and are adjusted for demographic characteristics, cardiac risk factors, disease severity on index presentation, comorbid diseases, and per capita concentration of cardiologists. AHR indicates adjusted hazard ratio; AMI indicates acute myocardial infarction.

Three years following the first AMI hospitalization, outcome gradients were largely preserved. Namely, the AHRs of all-cause death increased with progressively increasing marginalization (Q2 AHR, 1.13; 95% CI, 0.95-1.35; Q3 AHR, 1.25; 1.05-1.48; Q4 AHR, 1.35; 95% CI, 1.15-1.59; and Q5 AHR, 1.52; 95% CI, 1.29-1.80). Over 3 years, patients in the most marginalized quintile also had greater AHRs of hospitalization from all causes (AHR, 1.21; 95% CI, 1.15-1.27) and AMI (AHR, 1.20; 95% CI, 1.09-1.32) (Figure 3).

### Care Processes After Index AMI Hospitalization

In our cohort, rates of physician follow-up 30 days after the index AMI hospitalization were 68.7% for primary care physicians and 31.9% for cardiologists. We observed gradients in the proportion of patients with at least one physician visit or cardiac diagnostic test by neighborhood marginalization quintile (Table 2). Specifically, we noted lower rates of 30-day follow-up in the most marginalized quintile with primary care physicians (lower by 6.4 percentage points from Q1 to Q5) and cardiologists (lower by 10.5 percentage points from Q1 to Q5). The most marginalized patients were also less likely to receive an echocardiogram (lower by 3.3 percentage points from Q1 to Q5) or stress test (lower by 6.9 percentage points from Q1 to Q5) over 30 days. Across quintiles of marginalization, the proportion of patients who underwent repeat coronary angiography and revascularization were comparable. Patterns in the use of health services persisted over 1 year. Over 1 year, we observed differences between Q1 and Q5 in visits to primary care physicians (Q1, 96.1%; Q5, 91.6%) and cardiologists (Q1, 88.0%; Q5, 75.7%), as well as diagnostic testing (eTable 3 in Supplement 1).

**Table 2.** Health Service Utilization Over 30 Days by Marginalization Quintile

Characteristic	Patients by marginalization quintile, No. (%)				
	Quintile 1 (least marginalized) (n = 9654)	Quintile 2 (n = 10 916)	Quintile 3 (n = 13 030)	Quintile 4 (n = 15 899)	Quintile 5 (most marginalized) (n = 15 965)
Physician visits					
Primary care physicians	6956 (72.1)	7669 (70.3)	8958 (68.7)	10 926 (68.7)	10 483 (65.7)
Cardiologists	3651 (37.8)	3767 (34.5)	4111 (31.6)	4986 (31.4)	4362 (27.3)
Diagnostic testing					
Echocardiograms	1938 (20.1)	2040 (18.7)	2324 (17.8)	2769 (17.4)	2680 (16.8)
Ambulatory ECG monitoring	473 (4.9)	497 (4.6)	523 (4.0)	663 (4.2)	586 (3.7)
Stress testing	1791 (18.6)	1800 (16.5)	1922 (14.8)	2081 (13.1)	1874 (11.7)
Invasive evaluation and treatment					
Repeat coronary angiography	385 (4.0)	409 (3.7)	522 (4.0)	612 (3.8)	555 (3.5)
Revascularization	440 (4.6)	457 (4.2)	602 (4.6)	730 (4.6)	661 (4.1)

Abbreviation: ECG, electrocardiogram.

## Discussion

In this population-based study of younger AMI survivors, we found that progressively increasing neighborhood marginalization was associated with marked gradients in mortality and adverse outcomes that began shortly after hospital discharge and persisted or worsened over time. One year following hospital discharge, patients in the most marginalized quintile had hazards of death and hospitalization that were 80% and 20% higher, respectively, than patients in the least marginalized quintile. They were also less likely to be seen by primary care physicians and cardiologists despite universal coverage for medical and physician services being available in Ontario. Our findings indicate that younger patients living in marginalized neighborhoods in a universal health care system experienced significant disparities in post-AMI outcomes and care, suggesting that many barriers to care persist beyond financial access.

Many studies have established associations between socioeconomic status and outcomes after an AMI.^4,16,29,30,31,32,33,34,35,36,37,38,39,40,41^ Our study extends current knowledge in several ways. First, while the impacts of individuals’ socioeconomic status in cardiovascular outcomes is well established, few studies have examined the role of the neighborhood environment on AMI outcomes.^3^ Second, all patients included in our analyses received cardiac catheterization and survived to discharge, minimizing the chance that differences we observed in outcomes were impacted by disparities in baseline morbid status or care received during the initial hospitalization (eg, antiplatelets, revascularization). Third, while much of the literature considers in-hospital care and outcomes in jurisdictions permitting private payments for health services,^29,30,31^ our study provides insights into how younger patients use health services after leaving the hospital and their long-term outcomes, particularly in the absence of direct financial barriers to accessing physician services and cardiac testing.

Our findings of worse outcomes among more marginalized patients are aligned with international data showing associations of neighborhood socioeconomic disadvantage with mortality after AMI in younger patients.^3,18^ However, they diverge from prior work done by our group showing smaller long-term mortality gradients in older AMI survivors from marginalized neighborhoods (HR in Q5: 1.13 [95% CI, 1.03-1.22]).^42^ Neighborhood marginalization may mediate adverse impacts on health for younger individuals through multiple mechanisms. For example, younger individuals may be more likely to live in areas where aspects of the built environment (eg, walkability,^43^ proximity to roadways,^44^ and noise^45^ and air pollution^46^) have deleterious impacts on health behaviors such as physical activity levels. Additionally, marginalization may impact health outcomes by influencing the social environment. Neighborhood social cohesion has been associated with greater diffusion of health information, collective ability to advocate for health resources, as well as more social and psychological support.^47^ Older individuals may have also derived better adaptations over time to life stressors such as AMI and the impacts of the neighborhood environment on aspects of successful convalescence.

Our study also found that younger patients in the most marginalized quintiles were less likely to see physicians and received fewer cardiac diagnostic tests. These disparities were pronounced (absolute differences of approximately 10%) for rates of cardiologist visits over 30 days and worsened over time. These are important findings given that cardiologist care has been shown to be associated with lower mortality and adverse outcomes in chest pain^48^ and myocardial infarction populations^49^ as well as other cardiovascular conditions.^50^ The observed differences in health service utilization among marginalized patients warrant further investigation to better understand the underlying structural and systemic factors. While differences in geographical proximity to cardiologists and primary care physicians by marginalization quintile may explain some of the gradients seen in our study, we accounted for this by adjusting for the per capita supply of cardiologists. By contrast, patients from the most marginalized neighborhood quintiles in our cohort were more likely to be sole earners and to have dependents. This makes it possible that our findings reflect less visible barriers to accessing care such as the inability to arrange time away from work or substitute caregivers for children and elderly relatives. Marginalized individuals also experienced greater housing instability, which might have precluded longitudinal follow-up with health care professionals. Studies have shown that younger patients are more likely to be in the workforce at the time of an AMI, have fewer social supports, and are less likely to have prepared for adverse impacts of an AMI on employment and aspects of personal care. It is also possible that additional social factors (eg, language barriers, limited transportation) and medical factors (impaired mobility, mental health) further reduced follow-up care.^51^

Our results have important implications. Together, they suggest that AMI survivors from marginalized neighborhoods live shorter less healthy lives. They also indicate that younger individuals may be more vulnerable to the detrimental impacts of neighborhood marginalization^42^ and that supports beyond universal health care may be required to truncate inequities in access to care and outcomes. Inequitable distribution of resources and opportunities across neighborhoods may perpetuate inequities in cardiovascular health.^1^ From a policy standpoint, initiatives bolstering stable housing, paid sick leave, and caregiver support may allow for more physician appointments and participation in time-intensive investigations while decreasing emergent hospitalizations and premature death.^52,53,54,55^ These may be further complemented by interventions to decentralize postdischarge care, for instance, through nurse-led clinics^56^ and virtual care platforms.^57^ Finally, universal prescription drug coverage and income-raising interventions may reduce mortality by making life-saving medications more affordable for younger patients.^58,59,60,61^

### Limitations

This study bears notable potential limitations. First, while using an area-based metric allowed us to provide holistic insights into the influence of neighborhood socioeconomic factors on care and outcomes, it made it possible that we underestimated the risk to individual AMI survivors.^62,63^ Next, given the diverse composition of Ontario’s population, it is likely that sources of disparities observed in our study also include unexamined aspects of unconscious bias and structural racism.^1,64^ Additionally, we are unable to speak to some possible factors associated with excess mortality because we lacked information on race and ethnicity, household income, functional disability, mental health, and alcohol and other substance misuse. Further, while it is possible that differences in physician care may have resulted in greater hospitalizations, we did not test this formally through mediation analysis. Finally, the observational nature of our study makes it possible that differences we observed reflect unaddressed confounding. This is less likely, however, given that we selected a cohort of AMI survivors, all of whom received invasive evaluations. Also, we performed extensive adjustments for risk factors and other comorbidities.

## Conclusions

In this cohort study, despite comprehensive coverage for medical services, younger AMI survivors residing in marginalized neighborhoods had greater mortality and received less clinical care. Our findings suggest that younger individuals may be more vulnerable to the adverse effects of neighborhood marginalization and that universal health care may be insufficient to redress outcome inequities after AMI.

## Notes

Supplement 1. **eTable 1.** Dimensions of the Ontario Marginalization Index Used to Ascertain Neighbourhood-Level Marginalization **eTable 2.** Mortality and Adverse Events in Patients by Marginalization Quintile **eTable 3.** Health Service Utilization Over 1 Year by Marginalization Quintile **eFigure.** Adverse Events Within Thirty Days Of Index Acute Myocardial Infarction Hospitalization by Marginalization Quintile

Supplement 2.
Data Sharing Statement
