# Supplementary material for: Marginalized Neighborhoods and Health Outcomes in Younger Myocardial Infarction Survivors
Source: JAMA Netw Open. 2025 Jul 2;8(7):e2518826. doi: 10.1001/jamanetworkopen.2025.18826 (PMC12223890; doi:10.1001/jamanetworkopen.2025.18826)
Supplement: Supplement 1. — eTable 1. Dimensions of the Ontario Marginalization Index Used to Ascertain Neighbourhood-Level Marginalization eTable 2. Mortality and Adverse Events in Patients by Marginalization Quintile eTable 3. Health Service Utilization Over 1 Year by Marginalization Quintile eFigure. Adverse Events Within Thirty Days Of Index Acute Myocardial Infarction Hospitalization by Marginalization Quintile [file jamanetwopen-e2518826-s001.pdf]

Supplemental Online Content

Akioyamen LE, Sivaswamy A, Haldenby O, et al. Marginalized neighborhoods and health outcomes in younger myocardial infarction survivors. *JAMA Netw Open.* 2025;8(7):e2518826. doi:10.1001/jamanetworkopen.2025.18826

eTable 1. Dimensions of the Ontario Marginalization Index Used to Ascertain Neighbourhood-Level Marginalization

eTable 2. Mortality and Adverse Events in Patients by Marginalization Quintile

eTable 3. Health Service Utilization Over 1 Year by Marginalization Quintile

eFigure. Adverse Events Within Thirty Days Of Index Acute Myocardial Infarction Hospitalization by Marginalization Quintile

This supplemental material has been provided by the authors to give readers additional information about their work.

© 2025 Akioyamen LE et al. *JAMA Network Open*.

eTable 1. Dimensions of the Ontario Marginalization Index used to ascertain neighbourhood-level marginalization*

**Residential instability (also called households and dwellings)**	
	Proportion of the population living alone
	Proportion of the population who are not youth (age 5-15)
	Average number of persons per dwelling
	Proportions of dwellings that are apartment buildings
	Proportion of the population who are single/divorced/widowed
	Proportion of dwellings that are not owned
	Proportion of the population who moved during the past 5 years
**Material deprivation (also called material resources)**	
	Proportion of the population aged 20+ without a high-school diploma
	Proportion of families who are lone parent families
	Proportion of total income from government transfer payments for population aged 15+
	Proportion of the population aged 15+ who are unemployed
	Proportion of the population considered low-income
	Proportion of households living in dwellings that are in need of major repair
**Dependency (also called age and labor force)**	
	Proportion of the population who are aged 65 and older
	Dependency ratio (total population 0-14 and 65+/ total population 15 to 64)
	Proportion of the population not participating in labour force (aged 15+)
**Ethnic concentration (also called racialized and newcomer populations)**	
	Proportion of the population who recent immigrants (arrived in the past 5 years)
	Proportion of the population who self-identify as a visible minority

* The summary score of marginalization was derived by combining residential instability, material deprivation and, dependency and dividing by 3 to create quintiles.

© 2025 Akioyamen LE et al. *JAMA Network Open*.

eTable 2. Mortality and adverse events in patients by marginalization quintile

Characteristic	Q1 (least marginalized)	Q2	Q3	Q4	Q5 (most marginalized)
	N=9,654	N=10,916	N=13,030	N=15,899	N=15,965
**30 days**					
All-cause death	16 (0.2)	26 (0.2)	55 (0.4)	67 (0.4)	86 (0.5)
AMI hospitalization	276 (2.9)	310 (2.8)	383 (2.9)	466 (2.9)	524 (3.3)
All-cause hospitalization	616 (6.4)	724 (6.6)	893 (6.9)	1,147 (7.2)	1,247 (7.8)
**1 year**					
All-cause death	82 (0.8)	131 (1.2)	181 (1.4)	280 (1.8)	387 (2.4)
AMI hospitalization	435 (4.5)	504 (4.6)	642 (4.9)	804 (5.1)	919 (5.8)
All-cause hospitalization	1,704 (17.7)	2,010 (18.4)	2,644 (20.3)	3,382 (21.3)	3,672 (23.0)
**3 years**					
All-cause death	212 (2.2)	314 (2.9)	449 (3.4)	648 (4.1)	833 (5.2)
AMI hospitalization	654 (6.8)	805 (7.4)	958 (7.4)	1,247 (7.8)	1,450 (9.1)
All-cause hospitalization	2,755 (28.5)	3,306 (30.3)	4,161 (31.9)	5,471 (34.4)	5,879 (36.8)

Values are n (%), unless otherwise specified.
AMI – acute myocardial infarction.

© 2025 Akioyamen LE et al. *JAMA Network Open*.

eTable 3. Health service utilization over 1 year by marginalization quintile

Characteristic	Q1 (least marginalized)	Q2	Q3	Q4	Q5 (most marginalized)
	N=9,654	N=10,916	N=13,030	N=15,899	N=15,965
**Physician visits**					
Primary care physicians	9,273 (96.1)	10,376 (95.1)	12,209 (93.7)	14,841 (93.3)	14,620 (91.6)
Cardiologists	8,494 (88.0)	9,223 (84.5)	10,619 (81.5)	12,784 (80.4)	12,080 (75.7)
**Diagnostic testing**					
Echocardiograms	6,067 (62.8)	6,621 (60.7)	7,784 (59.7)	9,269 (58.3)	9,106 (57.0)
Ambulatory ECG monitoring	1,706 (17.7)	1,885 (17.3)	2,122 (16.3)	2,522 (15.9)	2,378 (14.9)
Stress testing	6,432 (66.6)	6,986 (64.0)	7,903 (60.7)	9,193 (57.8)	8,602 (53.9)
**Invasive evaluation and treatment**					
Repeat coronary angiography	1,205 (12.5)	1,410 (12.9)	1,729 (13.3)	2,100 (13.2)	1,984 (12.4)
All revascularization	964 (10.0)	1,071 (9.8)	1,393 (10.7)	1,704 (10.7)	1,578 (9.9)

Values are n (%).
ECG – electrocardiogram.

© 2025 Akioyamen LE et al. *JAMA Network Open*.

eFigure. Adverse events within thirty days of index acute myocardial infarction hospitalization by marginalization quintile

Outcome	Sample Size	Events (%)	Adjusted HR (95% CI)
All-cause death			
Q1	9,654	16 (0.2)	1.00 (1.00 to 1.00)
Q2	10,916	26 (0.2)	1.33 (0.71 to 2.48)
Q3	13,030	55 (0.4)	2.24 (1.28 to 3.92)
Q4	15,899	67 (0.4)	2.12 (1.22 to 3.69)
Q5 (most marginalized)	15,965	86 (0.5)	2.43 (1.41 to 4.18)
All-cause hospitalization			
Q1	9,654	616 (6.4)	1.00 (1.00 to 1.00)
Q2	10,916	724 (6.6)	1.06 (0.95 to 1.18)
Q3	13,030	893 (6.9)	1.08 (0.97 to 1.20)
Q4	15,899	1,147 (7.2)	1.08 (0.98 to 1.19)
Q5 (most marginalized)	15,965	1,247 (7.8)	1.16 (1.05 to 1.28)
AMI hospitalization			
Q1	9,654	276 (2.9)	1.00 (1.00 to 1.00)
Q2	10,916	310 (2.8)	1.02 (0.87 to 1.20)
Q3	13,030	383 (2.9)	1.04 (0.89 to 1.22)
Q4	15,899	466 (2.9)	1.00 (0.86 to 1.16)
Q5 (most marginalized)	15,965	524 (3.3)	1.12 (0.96 to 1.30)

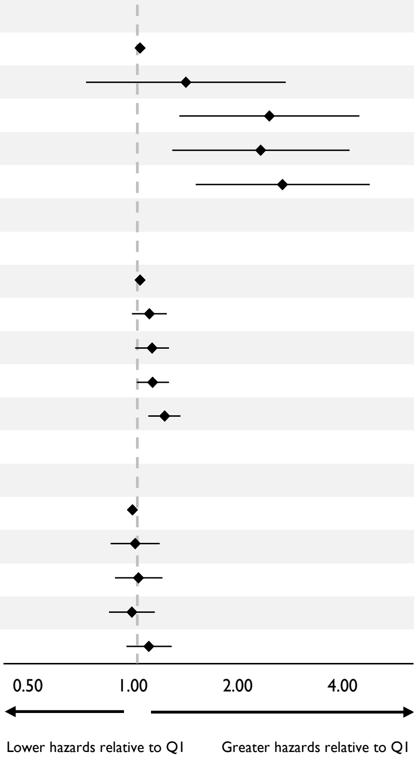

© 2025 Akioyamen LE et al. *JAMA Network Open*.
